# Analysis of Nurses’ Attitudes toward Patient Death

**DOI:** 10.3390/ijerph192013119

**Published:** 2022-10-12

**Authors:** Anna Maria Cybulska, Monika Anna Żołnowska, Daria Schneider-Matyka, Marta Nowak, Małgorzata Starczewska, Szymon Grochans, Aneta Cymbaluk-Płoska

**Affiliations:** 1 Department of Nursing, Pomeranian Medical University in Szczecin, Żołnierska 48, 71-210 Szczecin, Poland; 2 Department of Cardiology and Invasive Cardiology, Independent Provincial Public Integrated Hospital, Arkońska 4, 71-455 Szczecin, Poland; 3 Department of Clinical Nursing, Pomeranian Medical University in Szczecin, 71-210 Szczecin, Poland; 4 Department of Gynecological Surgery and Gynecological Oncology of Adults and Adolescents, Pomeranian Medical University in Szczecin, 70-111 Szczecin, Poland

**Keywords:** death, attitudes toward death, fear of death, dying, nurse

## Abstract

(1) The aim of the study was to analyze nurses’ attitudes toward a patient’s death, taking into account the emotions they experience and the general perception of death. (2) The study involved 516 nurses from the West Pomeranian Voivodeship in Poland. The research was carried out using the diagnostic survey method using The Death Attitudes Profile Revisited (DAP-R-PL), the Scale of Fear and Fascination with Death, and a demographic questionnaire. (3) Research has shown that nurses accept the phenomenon of death as a natural process of human life; however, they adopt the attitude of fear of death. Most of the respondents experienced: sadness (73.4%), helplessness (58.5%), and regret (43.6%) due to the patient’s death. (4) Both age, sex, marital status, and place of residence significantly influenced the attitudes of nurses toward the patient’s death. Therefore, it is important to provide psychological support or special education in the case of dealing with the fear of death.

## 1. Introduction

Attitudes toward death are varied, and a person shapes his attitude toward death depending on what elements of its object he perceives and how he experiences them. In the case of death, it can be concluded that the differentiation of the attitude toward death is conditioned by the complexity of the phenomenon of death and the uniqueness of the human personality [1]. Thinking about death is a cognitive element of human activity and allows you to be conscious of meeting reality. Death anxiety is a multidimensional construct with cognitive, emotional, and experiential attributes [2]. It is important to distinguish thinking about death from fear of death. The very contemplation of death is closely related and results from a certain reflection and distance to the phenomenon of death, while fear is an emotional reflex [3]. The attitude toward death may be a consequence of the adopted philosophy of life, religion or style of behavior in terms of culture [3]. A characteristic feature of the attitude toward death is the fear of death and fear or anxiety related to death. Fear is a reaction to an apparent (objectively existing) danger, while anxiety is a reaction to a hidden (subjective) danger. Thus, fear means an emotional reaction without signs of pathology, while fear is perceived as an objectless reaction, not externally conditioned, and a sign of pathology [4]. The fear of death has specific origins. They are primarily located in biological human instincts and species behavior and are related to the subconscious struggle of man for his own life. Such an active attitude toward one’s own destiny is therefore written deeply in consciousness. Indifferent and passive attitudes are extremely rare [5].

Death is a part of natural life. Moreover, it is a psychological as well as a physical event that affects the person who is dying and also the people caring for that person, especially health professionals [6]. The nurse, whose task is to take care of the patient, often has the privilege of accompanying patients in the process of dying and the process of death itself. In the Code of Professional Ethics of the Polish Nurses and Midwives, there is a provision that says that a nurse and a midwife should strive to provide the ward with dignified conditions for dying while respecting the values they recognize [7]. Providing decent conditions at the last stage of a patient’s life concerns one of the most important moments in a professional nurse’s work [8].

Death is always a stressful experience, even when it comes to a stranger. However, it should be remembered that nurses often become emotionally attached to their patients, and their departure always remains deep in their memories. The nurse in the team is the person who accompanies the physically, mentally, and spiritually suffering person and their relatives on a daily basis. Providing the best possible quality of life for a patient with hope is a key role of the palliative and hospice nurse. She creates nursing open to other people, in which it is necessary to stop at the dying person but also to provide support and help to the family during illness and orphanhood [9].

Proximity to death and to the patient’s suffering can influence the emotional status of healthcare workers. They can feel fears, anxieties, distress, grief, failure, and frustration. Taking care of dying patients can be an emotionally painful, stressful, and distressing experience. As we know, most of the research concerns the assessment of nurses’ attitudes toward the death of a patient. Much less frequently, these studies are carried out among other healthcare professionals. Awareness of the processes of dying and coping with death are important, especially for nurses, since accompanying the dying and coping with death are part of their daily work. The emotional management skills of healthcare professionals have a direct impact both on the quality of end-of-life care they can provide and also on their own well-being [10]. 

Nurses in the workplace are exposed to severe stressors because they are emotionally involved in the psychological, social, and physical problems of their patients. Nurses often have effective relationships based on emotional bonding with patients. Work-related stress has a negative impact on many aspects of nurses’ lives and contributes to many health complications and to burnout syndrome [11]. The fear of death is also associated with the development of many mental disorders [12]. Fear of death may induce adverse reactions to the care of dying patients [13] or be associated with negative health effects, such as exposure to life-threatening events, mental stress, dissatisfaction with life or deterioration of physical functions [14]. Some studies have shown that nurses have a greater fear of death and can be more vulnerable to the destructive influence of negative emotions than other healthcare workers [15,16]. Nurses may choose strategies to protect themselves from these emotions, such as denial of feelings, which can be a defense mechanism for fighting the effects of exposure to the dying process, allowing the daily functioning of the healthcare workers.

The nursing profession belongs to the group of extremely stressful professions, burdened with many stressors, the most important of which are the responsibility for the health and life of patients and the death of patients. Psychological skills that predispose to this profession are, therefore, closely related to the appropriate personality predispositions and the ability to deal with stress, which is extremely useful in a difficult or crisis situation in which you need to make the right decision quickly. The work of a nurse is physically and mentally demanding, mainly due to the interpersonal nature of this work, i.e., being between people and experiencing their tragedies due to the shift in work. Moreover, high social demands are placed on nurses, which implies the emergence of specific tensions, fears or even aggression in this professional group. 

Death has a great emotional impact on nurses [17]; therefore, resilience can be one of the key emotional competencies for managing anxiety around death and dying. Training to promote the development of resilience would constitute a way to improve the quality of care [18], enhance well-being [19], and improve care practice [20], but it can also increase psychological health [21].

The review showed that attitudes toward death in nursing professionals are connected to several sociodemographic, personal, and training factors. Caring for dying patients is especially difficult and demanding for nurses, making them face their own death and increasing their feelings of anxiety [22]. Importantly, life experiences with death may also impact attitudes toward death and contribute to levels of death anxiety [22]. Furthermore, death anxiety brings about important behavioral and emotional consequences [23].

To our knowledge, there are no standardized programs included in undergraduate nursing degrees focused on increasing resilience or providing skills to cope with caring for dying patients. There are studies concerning resilience and its role in promoting the well-being of healthcare workers [24], but there is little data on how it may protect against the stresses and anxieties that accompany end-of-life care. Moreover, nurses are more vulnerable to stress related to direct, intense, and prolonged contact with dying patients. Therefore, it is important to understand nurses’ attitudes toward death because a better understanding of nurses’ attitudes, may ultimately lead to interventions that can offset serious consequences such as poor patient care, decrements in personal health, and burnout.

In order to address these gaps in the literature, our study will analyze the nurses’ attitudes toward a patient’s death, taking into account the emotions they experience and the general perception of death.

We hypothesized that:

- Nurses accept death as part of life and do not feel fear of death.
- Sadness, helplessness and regret are the most common types of nurses’ emotions caused by the death of patients, regardless of the nurses’ length of service and the place of work

We formulated the following research questions:

- What are nurses’ most common attitudes toward death and the sociodemographic, professional, factors that significantly affect those attitudes?
- How does a patient’s death influence an approach to life among nurses?

## 2. Materials and Methods

### 2.1. Participants

The research involved 516 nurses, where the vast majority of respondents (93.6%) were women, married (53.7%), living in cities with more than 100,000 inhabitants (35.1%), with a bachelor’s degree (51%), and persons declaring work experience up to 5 years (39.1%). Based on the collected data, it was shown that 77.8% of the respondents worked in a hospital, of which 67.6% were employed only in one workplace, and 80% worked in a two-shift system (Table 1). 

### 2.2. Instruments

The study used the diagnostic survey method, the original questionnaire was used, which consisted of 21 questions concerning sociodemographic data (age, sex, marital status, place of residence, education) and selected aspects related to the patient’s death, and standardized research tools were used.

The Death Attitudes Profile Revisited (DAP-R-PL) in a revised version by P.T.P. Wong, G.T. Reker, G. Gesser was used in this study. The questionnaire consists of 32 statements relating to different attitudes toward death. The comparison of the average results obtained in the individual scales of the DAP-R-PL questionnaire for the entire surveyed group of respondents makes it possible to determine the dominant attitude toward death in the studied group. Questionnaire scales: fear of death; death avoidance; natural acceptance of death; theological acceptance of death; and escape avoidance. Each statement is scored from 1 to 7, depending on the answer chosen. The arithmetic mean is calculated from the total points obtained for all questions in a given scale from all respondents, and the obtained value is a measure of the frequency of occurrence of a given attitude in the studied group. A comparison of the obtained indicators for a given scale indicates which attitude out of the five included in the questionnaire dominates among the respondents. The results can be related to the entire study population and its individual groups divided by sex, age, place of residence, etc. Cronbach’s alpha coefficient for the DAP-R-PL five factors, ranged between α =  0.76 (Escape Acceptance) and α = 0 .86 (Approach Acceptance). The scale with the lowest reliability index was Neutral Acceptance (α =  0.56) [25].

The Scale of Fear and Fascination with Death (Żemojtel - Piotrowska M., Piotrowski J.) is a measurement method that checks the relationship between fear and fascination with the phenomenon of death. This questionnaire consists of two subscales: fear of death and fascination with death. The fear of death subscale is used to measure the general fear of death, especially in relation to oneself, notwithstanding the differentiation between the fear of dying, the fear of suffering, or the final separation from loved ones. The subscale of fascination with death measures both cognitive fascination with the subject of death and dying, as well as acceptance of the possibility of a person committing suicide, together with the declared death wish. Each statement is scored from 1 to 4. The arithmetic mean is calculated from the total points obtained for all questions in a given scale from all respondents, and the obtained value is a measure of the frequency of occurrence of a given attitude in the study group. A comparison of the obtained indicators for a given scale indicates which attitude out of the five included in the questionnaire dominates among the respondents. The results can be related to the entire study population as well as its individual groups divided by sex, age, place of residence, etc. Cronbach’s alpha coefficient for the “Anxiety” subscale was 0.80, and for “Fascination” it was 0.90 [26].

### 2.3. Procedure

The study involved 516 nurses from the West Pomeranian Voivodeship in Poland. The criterion for inclusion in the study was: having the right to practice as a nurse and active performance in the profession. Potential respondents were invited electronically to take part in the survey, which was conducted in accordance with the principles contained in the Helsinki Declaration. Participation in the study was voluntary and anonymous, and respondents were informed about the possibility of withdrawing from participation in the study at any stage.

### 2.4. Ethical Aspect

All procedures performed involving human participants were in accordance with the ethical standards of the institutional research committee and with the Helsinki declaration.

Ethical review and approval were not required for the study with human participants in accordance with the local legislation and institutional requirements. Our study was conducted taking into account ethical considerations. Informed consent was required, and participation in the study was voluntary. Moreover, the participants were assured of anonymity and confidentiality and were free to withdraw from the study at any moment.

### 2.5. Data Analyses 

The obtained results were statistically analyzed with the use of Microsoft Excel 365 (Microsoft Corporation, Albuquerque, NM, USA). The Pearson correlation coefficient was used to determine the level of linear dependence of two variables, and the degree of dependence of two measurable variables is <−1.1>. A value equal to 0 means there is no linear relationship between the features, while a value closer to 1 or −1 means a stronger value. A *p*-value of <0.05 was considered statistically significant.

## 3. Results

### 3.1. Analysis of Factors Related to the Functioning of Nursing Staff That Affect the Attitude of Medical Personnel toward Death

The study analyzed the memory of the first patient’s death. The vast majority of respondents (77.5%) remembered the first patient who died during their on-call time. In turn, 18.8% of respondents said that they do not remember this situation anymore, and 3.7% have never experienced such a situation.

Moreover, the self-awareness of nurses toward their own emotions and feelings during the patient’s death was analyzed. Most of the respondents experienced: sadness (73.4%), helplessness (58.5%), and regret (43.6%) due to the patient’s death. Among other emotions, the respondents mentioned: anxiety (26.6%), feeling of injustice (21.1%), and feeling of being a witness to a secret (17.8%). The least frequently respondents felt embarrassment (1.7%) and fascination (1.7%).

In response to the issues related to the factors influencing the perception of the patient’s death by nurses, it was observed that the age of the patient was the dominant factor (85.5%). Moreover, 68.0% of the respondents made their attitude toward the patient’s death dependent on the type of death (sudden, slow), while 56.6%—on the relationship with the patient. In turn, 21.1% of the respondents indicated that the attitude toward the death of the patient might depend on the relationship with his family. In the case of 4.8% of the respondents, the perception of the patient’s death was influenced, among others, by the type of illness of the patient, the religious beliefs of nurses, and the life experiences of the patient.

The research analyzed the opinions of nursing staff about the preparation for the death of the patient. Almost half of the respondents (49.8%) believed that it was possible to prepare for the death of a patient, while 39.3% of the respondents believed that it was not possible. A total of 10.9% of the respondents had no opinion in this regard. The vast majority of respondents (76.7%) did not feel the need for support after the patient’s death. Among people who expected such support (23.3%), they most often looked for it among family members (58.7%) or among nurses working in the same ward (47.1%). Significantly fewer people needed the help of a psychologist (16.9%) and support from the ward nurse (4.4%).

Factors influencing better coping with the patient’s death were verified. The vast majority of respondents believed that talking about death in the workplace (56.4%) and longer work experience (52.1%) had a positive impact on coping with the patient’s death. Among the remaining respondents, contact with death (46.5%), the help of a psychologist (28.3%), and the use of relaxation methods (22.5%) contributed to better coping with the patient’s death. The remaining respondents (4.8%) believed that the ability to separate work and private life, faith in God, life experiences, training, or individual approach to death might play a key role in accepting the patient’s death.

### 3.2. Nursing Staff Attitudes Analysis toward the Patient’s Death

The standardized Attitude Profile to Death (DAP-R-PL) questionnaire was used to assess the attitudes of nursing staff toward the patient’s death. The research results were analyzed taking into account: fear of death, avoidance of death, natural acceptance of death, theological acceptance of death, and escape acceptance of death.

Based on the collected data, it was observed that the respondents fear death to a large extent. The vast majority of respondents agreed with the statements that death is an unpleasant experience (63%), the prospect of my own death arouses anxiety (46.9%), death means the end of everything that scares me (35.3%), and that death is unavoidable, which makes them anxious (34.1%). In the case of the following statements: I am very afraid of death, the subject of life after death worries me a lot and I am worried about the lack of certainty about what will happen after death, in most cases, the respondents did not have an unambiguous opinion on this subject (Table 2).

The vast majority of respondents did not exhibit an attitude of avoiding death. In the case of the following statements: whenever there is a thought about death in my head, I try to push it away, and I try not to think about death. Most of the respondents agreed with these statements. In the remaining cases, the respondents disagreed with them (I completely avoid thinking about death, and I try not to have anything to do with the subject of death, or they did not take any position (avoiding thoughts about death at all costs) (Table 2).

On the basis of the collected data, it was observed that the respondents naturally accepted death. The vast majority of respondents agreed with the statements: death should be perceived as a natural, undeniable and inevitable event (55.4%), death is a natural aspect of life (77.7%), I am not afraid of death, but I do not wait for it (39 %), and death is part of life as a process (67.8%). Only in terms of determining whether death is good or bad did the respondents not take a position (Table 2).

In the theological scale of acceptance of death, the respondents did not take a position in the case of nine out of ten statements. They only agreed that they expected a reunion with those they loved after death. The remaining statements, including, among others, faith in heaven, faith in a better place after death, transition to a better world, or freeing the soul, were found by the respondents to be difficult to define unequivocally (Table 2). The last attitude presented in the questionnaire is escape acceptance of death. The vast majority of respondents did not take any position on the statements: death will end all my problems, it is an escape from this cruel world, it is a release from earthly suffering, or I perceive death as a release from the burden of life. On the other hand, the respondents agreed with the statement that death is salvation from pain and suffering, and I perceive death as a release from earthly suffering (Table 2 and Table S1).

The analysis of the data obtained from the Questionnaire Profile of the Attitude Toward Death (DAP-R-PL) showed that in the case of the scales of fear of death and natural acceptance of death, the respondents obtained the highest score, i.e., 5.3 points. This means that the respondents display mainly these two attitudes toward the patient’s death. Slightly lower scores were obtained in the theological acceptance of death (4.6 points) and in escape acceptance of death (4.4 points). The lowest score, i.e., 3.9 points, was obtained by the respondents in the case of avoiding death (Table 3).

#### 3.2.1. Assessment of the Level of Anxiety and Fascination with Death among Nurses

In the studies, the Scale of Fear and Fascination with Death was used to assess the level of anxiety and fascination with death among nurses. The research results were analyzed taking into account: fear of death and fascination with death.

On the basis of the obtained results, it was observed that the respondents did not show any fear of death. Most of the respondents agreed with the statement: “thinking about death depresses me” and “the prospect of my death is terrifying to me”. In the case of the remaining statements, the respondents strongly negated them. They included such issues as: “I like to imagine how I will die”, “in moments of severe depression I start to think about my death”, “in a really difficult situation I could decide to commit suicide”, “I think about death without fear”, “I don’t like movies in which one of the main characters dies“, “sometimes it is difficult for me to distract myself from the thoughts of death”, “sometimes I imagine what will happen to me after death” (Table 4).

The respondents adopted an attitude that negated their fascination with death. Most of the respondents agreed with the statement: “death is something unpleasant for me”, “I think that I will pray more as the years go by”, and “it fills me with horror when people close to me die”. However, the vast majority of claims have been denied. They included such phrases as: “I often wonder in what situation I would be willing to take my own life”, “when someone raises the issue of death in my presence, I quickly try to change the topic of conversation”, “I often think about death”, “I like to imagine my own funeral”, “I often think that I would like to die”, “it terrifies me when people close to me die”, “death is a fascinating mystery to me”, "death is the worst misfortune in human life”, “I like to learn about various rituals and ceremonies accompanying death”, “I like to meditate on topics related to death”, “I am interested in pictures of death and dying”, and “I often talk to people about death” (Table 4).

The analysis of the data obtained from the Scale of Fear and Fascination with Death showed that both in the case of fear of death and fascination with death, the respondents obtained an average score of 2.1 points, which was a result slightly above the average. This means that there is no clearly manifested attitude in the study group, and the result does not allow for an unambiguous indication of whether the above-mentioned attitudes are present in any way in the study group.

#### 3.2.2. Analysis of Sociodemographic Variables Influence on Attitudes toward the Patient’s Death Represented by Nurses and on the Level of Fear and Fascination with Death

The study analyzed the impact of selected socio-demographic variables (age, sex, marital status, place of residence, education) on attitudes toward the patient’s death and on the level of anxiety and fascination with death among nurses.

Based on the collected results, statistically significant correlations between age and two attitudes toward death were demonstrated: avoidance of death (r = −0.2147) and natural acceptance of death (r = 0.3412). Younger respondents more often have the former attitude toward death, while the elderly have the latter. Moreover, a statistically significant correlation was observed between gender and the fear of death (r = 0.2487) and the natural acceptance of death (r = −0.2637). It has been observed that women adopt an attitude of natural acceptance of death, and men fear death. In the case of marital status, a statistically significant correlation was demonstrated with the natural acceptance of death (r = 0.2875), and in the case of the place of residence, a significant correlation was observed with the theological acceptance of death (r = −0.2206) and escape acceptance of death (r = 0.4213). Inhabitants of villages more often adopted the attitude of theological acceptance of death, while inhabitants of larger cities escape the acceptance of death. No statistically significant correlation was observed between education and attitudes toward death, according to the DAP-R-PL questionnaire (Table 5).

In the case of any of the presented sociodemographic factors, no statistically significant correlations were found between the fear of death and the fascination with death (Table 5).

The analysis also covers the impact of seniority on attitudes toward death represented by nurses, as well as the level of fear and fascination with death.

On the basis of the collected data, a weak correlation was observed between the length of service and the escape acceptance of death. There was no correlation between work experience and the Scale of Anxiety and Fascination with Death (Table 6).

## 4. Discussion

Death is a phenomenon that evokes strong emotions, which is why it is a difficult experience for every human being. It touches the dying person and the people who accompany him in this process in a special way. Caring for the sick, communing with death, and often close relations with the patient and his family make a special relationship between the dying person and the caregiver. As a result, it may be difficult to cope with the patient’s death, and therefore the nursing staff incurs a specific emotional cost. Despite frequent accompanying dying patients, people working in medical professions are often unable to cope with the patient’s death; therefore, they exhibit different attitudes toward death, ranging from fear to the natural acceptance of death.

### 4.1. Factors Influencing the Perception of Death

Death and the dying process are one of the most stressful experiences for nurses. The feelings experienced by nurses are a reason for reflection, as there are moments that fall deeply into a person’s memory. 

According to the results of our own research, the patient’s death significantly affects the functioning of the nursing staff. The vast majority of respondents remembered the patient’s first death. The most common emotions felt were sadness, helplessness, and regret. In the opinion of nurses, the attitudes toward death they represent are influenced by: the patient’s age, type of death, and the relationship with the patient and his family. Nurses are most affected by the deaths of young patients they know well.

Many studies have confirmed that nurses experience a high level of stress and strong emotions triggered by the observation of dying patients. Moreover, the attitudes toward death in nursing professionals are connected to several sociodemographic and personal factors. 

The research by Cybulska et al. [27] showed that in the subjective opinion, the dominant type of attitude toward the death of a patient among the respondents was the emotional type (78%). An attitude of distancing themselves from death was declared by 22% of the respondents, and as many as 85% of the respondents remembered the first death of the patient. 

A study by Gołębiak et al. [28], for 50.8% of nurses, the patient’s death was an emotional situation, where the type of emotions and their intensity depended on the patient. The remaining nurses declared their approach to the patient’s death at a distance. The vast majority of respondents felt compassion, regret, and powerlessness.

In a study by Abu Hasheech et al. [29], it was shown that work experience and age are related to the attitude toward death and dying patients. The greater the experience, the more often the attitude of natural acceptance of death or escape acceptance of death is manifested. Nurses with 1–5 years of work experience more often displayed an attitude of fear of death or an escape from acceptance of death. The research corresponds with the obtained results of our own research.

On the other hand, studies by Barbosa and Vieira [30] showed that there is a strong correlation between the age and gender of nurses and the attitude toward the patient’s death. Older nurses showed a much higher escape acceptance of death, and the female gender favored the theological acceptance of death. Moreover, a relationship was demonstrated between the level of education and the runaway and theological acceptance of death. Nurses from palliative care facilities showed a lower level of fear of death compared to the rest of the surveyed nurses.

The research by Nyklewicz and Krajewska-Kułak [31] showed that among nurses, there was a strong fear and depression at the thought of their own death or the death of a loved one, while 52% of nurses experienced such emotions in connection with the patient’s death. Among these nurses, 75% experienced low or moderate anxiety, and the remaining 25% had high or very high anxiety. 

Many studies showed the lack of preparedness among student nurses for end-of-life care and the lack of support for nurses after patients’ death [28].

The authors’ own research has shown that most nurses believe that it is possible to prepare for a patient’s death. Moreover, in their opinion, they are prepared for this difficult situation and do not need any support after the patient’s death. It is worth noting, however, that nurses believe that the death of a patient may have an impact on the quality of their work, especially during on-call duty, during which the patient died. In addition, nurses who declare the need for support after the patient’s death mainly look for it in conversation with relatives, try not to think about the situation, pray, or cry. According to the nurses, a longer length of service allows them to cope better with the death of their patients.

On the other hand, in the study of 178 nurses and 25 male nurses working in a university hospital, the highest score in terms of fear of death was identified among respondents working in internal medicine clinics, and the lowest among ambulance nurses [32]. 

A study by Gołębiak et al. [28], conducted on a group of 115 nurses, showed a significant, directly proportional relationship between work experience and the self-esteem of the level of empathy toward a patient dying from nurses. The research of Gołębiak et al. also indicates that nurses often deal with the death of patients (48.7% of indications); therefore, nurses indicate that they need training in competencies and conditioning specialist preparation for the death of a patient. 

In the case of research conducted among 213 students of medical faculties, it was shown that students are not able to express their opinion on the degree of their preparation for caring for a dying patient. It was observed that 43.2% of the respondents indicated that they were not prepared to deal with a dying patient, and 64.3% of the respondents said that they were not prepared to care for a dying patient, mainly in terms of coping with emotions. For 55.9% of students, the most important thing would be to learn to communicate with the dying patient and his family (72.3%) [33].

Similar results were obtained by Jors et al. [34], who observed that medical and nursing staff from oncology departments needed training in knowledge and skills in palliative care, communication with a dying patient, and cooperation with his family. Moreover, studies conducted on a group of medical students showed deficiencies in communication with dying patients [35]. In turn, Pawłowski et al. [36] noted that after a palliative medicine course, 76% of medical students admit that they cope better with a dying patient.

Karadag et al. [37] observed that nurses who do not have a positive view of death and do not accept that death is a part of life could have negative emotions and behavior.

The study by Anderson et al. [38] showed that nurses who find death and dying very difficult might feel helplessness, insufficiency, distress or defensiveness, and coping mechanisms, for example, prevention, being distant, and avoidance.

Moreover, healthcare providers may have negative feelings such as grief, depression, despair, fear and anxiety, and guilt and have negative attitudes toward death [39,40]. A study by McKenzie et al. [41] showed that death anxiety has a negative influence on the capacity of nurses to carry out their roles effectively.

A review of the literature reported that factors such as age, gender, marital status, occupation, professional experience, witnessing someone’s death, beliefs, and coping strategies could influence people’s perception of death, the meaning of death and life, attitudes, and behaviors related to death and the level of death anxiety [40,41,42].

### 4.2. Nursing Staff Attitudes Analysis toward the Patient’s Death

The authors’ own research has shown that there is no single, dominant attitude of the surveyed group of nurses toward the patient’s death. It was identified that nurses fear death the most while treating it as a natural part of life and showing a natural acceptance of death. In the study group, there are also theological (philosophical) attitudes toward death, linking it with a better life, connection with God and meeting with loved ones, as well as a fleeing attitude toward death, when death is not avoided but rather as an escape from death.

Alternatively, in the study on attitudes accompanying paramedics during contact with death, three types were identified: neutral, distant, and emotional. According to the researchers, the elements of the ideal attitude adopted by paramedics toward death are of a neutral type [43]. On the other hand, in the study by Krajewska-Kułak et al. [44], death was accepted as a natural phenomenon by 41.2% of the surveyed nurses, including people with the shortest work experience (80%).

In studies using the DAP-R scale, principal component analysis revealed a five-factor solution, including fear of death, avoidance of death, natural acceptance of death, theological acceptance of death, and escape acceptance of death. Internal consistency for each subscale ranged from 0.64 to 0.88. Correlations between the DAP-R subscales supported the relative independence of the dimensions of attitudes toward death. According to the authors, the DAP-R scale can be used as a research and clinical tool to assess attitudes toward death among Greek nurses [45].

Meanwhile, a study of 357 Chinese nursing trainees using the DAP-R questionnaire identified that among the five domains of attitude toward death, the highest score was obtained in the domain of its natural acceptance, which means that most of the respondents considered death a natural phenomenon in human life. In addition, trainees with education about death scored higher in the domain of natural acceptance against death fear, death avoidance, and escape acceptance of death. The lowest result was obtained in the domain of theological acceptance of death, which may be related to the fact that only 9% of respondents declared religious beliefs [46].

A study assessing nurses’ attitudes toward patient death, before and after conducting psychological workshops was examined. Based on the collected data, it was shown that there was a significant improvement in the knowledge of nurses (*p* < 0.01) and a reduction in their fear of death index (*p* < 0.01). In addition, the scores for fear of death (*p* = 0.025) and death avoidance (*p* = 0.047) were significantly reduced. No changes were observed in the natural and runaway acceptance of garbage. After the workshops, more nurses adopted the attitude of neutral acceptance of death (76.2%), and none of them showed fear of death. Most of the surveyed nurses reported a positive change in their knowledge and attitudes toward death after the workshops [13].

Studies by Duran et al. [47] showed that the attitudes of nurses toward death were positive, and their fear of death was low. Moreover, approach acceptance was high in younger ones. A statistically significant positive correlation was found between nurses’ anxiety level and escape acceptance score. There was a statistically significant positive correlation between the length of working years of nurses and the escape acceptance and approach acceptance. 

Barnett et al. [48] found that hospice nurses had relatively low levels of fear of death and death avoidance and relatively higher levels of neutral acceptance, approach acceptance and escape acceptance.

Nursing is one of the most stressful jobs in which caring for a dying patient is an integral part. Very often, during studies, nursing students experience the death of a patient for the first time. In addition, during the nursing apprenticeship, students observe the dying process without receiving sufficient training regarding how to deal with crisis situations. Therefore, it is important to understand what nurses face in their work and provide them with the psychological and emotional support they need, taking into account organizational and environmental factors for better management of patient death. Moreover, during studies, teachers and mentors should provide adequate support and prepare students for contact with a dying patient. Persons responsible for designing training for nurses should include in their program assessment of attitudes toward death and interventions influencing the adoption of positive attitudes toward death. Nurses should engage in anti-stress therapies and therapies that allow them to maintain emotional resistance, thus reducing the risk of burnout syndrome. Additionally, employers should focus on removing or minimizing modifiable factors (e.g., workload, time pressure, staff shortages) and thus reduce the stress experienced by nurses. This will improve the quality of care and increase patient satisfaction and safety.

## 5. Limitations

The limitation of the study is the number of respondents—it does not represent the value of a representative professional group of nursing personnel. In addition, random group selection may affect the results. Another limitation is the method of obtaining data. The research technique used, although it is anonymous, poses a risk of obtaining false answers, which may result from conscious or subconscious manipulation of the answers by the respondent. The next limitation of the study is the fact that representatives of both sexes did not create groups of similar size, which may contribute to bias in the results, translating into the quality of the study and its credibility. In addition, our study did not take into account all variables that could significantly affect nurses’ attitudes toward death. We used questionnaires that did not include questions such as religious beliefs, death of a loved one, personal anxiety, the severity of stress, burnout, frequency and severity of job stress, mental health assessment, quality of life, and self-efficacy. However, the presented limitations do not disqualify the results of the study because, in the studies of other authors on the same subject, the number of the studied material, the method of obtaining the results, and the number division by sex were similar.

## 6. Conclusions

The death of a patient is a phenomenon that causes a strong reaction; therefore, most nurses remember the first contact with a patient’s death. The main emotions that accompany this situation are sadness, regret, and helplessness. Nurses show an attitude of fear toward death and its natural acceptance. Both the patient’s age, the type of death, and the relationship with the patient significantly affect the attitudes toward death adopted by nurses. Age, sex, marital status, place of residence, education, and professional experience of nurses also influence the attitudes of nurses toward the death of a patient. Therefore, it is important to provide nurses with significant psychological and emotional support and to take into account organizational and environmental factors for better management of the patient’s death.

## 7. Implications for Practice

This study highlights the need for further research and development of educational programs to help nurses to explore and understand their attitudes toward death, overcome fears, and enhance coping strategies. Adequate professional education and training about the attitude toward death are necessary starting from the undergraduate level. Nurses should be prepared to cope with death and supported on death with in-service training. It will be useful to provide these training programs to nursing students during their education process.

## Figures and Tables

**Table 1:** Nurse characteristics.

Sociodemographic Variables (*N* = 516)		*n*	%
Gender	Female	483	93.6
	Male	33	6.7
Age group [years]	<30	175	33.9
	31–45	150	29.1
	46–60	180	34.9
	>60	11	2.1
Marital status	Single	118	22.9
	Casual relationship	70	13.6
	Formal relationship	277	53.7
	Divorcee	36	7
	Widow	15	2.8
Place ofresidence	Village	149	28.9
	Town with a population 10–100 thousand	186	36
	Towns with a population over 100 thousand	181	35.1
Education	Secondary/ post-secondary education	71	13.8
	Bachelor of nursing studies	263	51
	Master of nursing studies	182	35.2
Seniority [years]	<5	202	39.1
	6–10	41	7.9
	11–15	28	5.4
	16–20	44	8.5
	21–30	124	24
	>30	77	14.9

*n*—number. %—percent.

**Table 2:** Partial results of the DAP-R-PL scale [%] (N = 516).

Item		Strongly Disagree	Don’t Agree	Rather Disagree	Hard to Say	Rather Agree	Agree	Strongly Agree
Fear of Death	DAP- 1	1.7	2.3	3.7	11.4	17.8	34.1	28.9
	DAP- 2	2.7	4.5	7.9	15.3	22.7	32.4	14.5
	DAP- 7	5.8	13.8	12.0	14.3	20.0	22.1	12.0
	DAP- 18	8.3	16.9	16.7	22.7	18.0	11.4	6.0
	DAP- 20	7.8	22.5	19.6	29.1	10.5	8.7	1.9
	DAP- 21	3.7	13.6	7.8	21.3	18.4	24.4	10.9
	DAP-32	2.7	10.3	9.9	23.8	20.3	21.9	11.0
Death Avoidance	DAP- 3	7.6	18.2	17.1	21.1	14.3	15.3	6.4
	DAP- 10	5.2	15.9	16.1	17.8	21.1	16.1	7.8
	DAP- 12	4.8	14.3	11.0	14.0	21.9	25.6	8.3
	DAP- 19	8.7	25.2	19.4	18.4	12.2	12.4	3.7
	DAP- 26	8.7	23.6	25.6	20.3	12.6	6.4	2.7
Neutral Acceptance	DAP- 6	0.6	1.7	3.5	12.0	26.7	33.5	21.9
	DAP- 14	1.2	0.2	0.2	4.3	16.5	46.5	31.2
	DAP- 17	4.7	7.6	11.4	20.3	17.1	26.2	12.8
	DAP- 24	0.2	1.0	2.1	8.7	20.2	44.4	23.4
	DAP- 30	0.8	5.4	4.5	40.1	16.7	24.0	8.5
Approach Acceptance	DAP- 4	5.6	5.8	4.7	41.7	16.3	16.1	9.9
	DAP- 8	8.7	9.9	6.6	50.4	10.9	9.5	4.1
	DAP- 13	5.6	6.4	2.5	43.0	15.3	15.7	11.4
	DAP- 15	5.4	3.7	5.0	35.7	17.6	21.3	11.2
	DAP- 16	4.5	6.0	5.2	45.9	15.5	14.7	8.1
	DAP- 22	2.7	1.6	2.1	20.3	18.6	33.3	21.3
	DAP- 25	4.3	4.7	3.3	38.8	19.8	20.5	8.7
	DAP- 27	3.3	3.1	3.7	43.8	16.5	22.9	6.8
	DAP- 28	5.0	5.6	7.4	26.9	22.3	19.6	13.2
	DAP- 31	4.1	3.9	4.7	35.1	18.8	23.3	10.3
Escape Acceptance	DAP- 5	9.7	8.9	7.6	28.1	14.3	20.3	11.0
	DAP- 9	12.0	22.3	12.8	28.9	13.6	6.4	4.1
	DAP- 11	3.5	4.1	3.3	17.4	27.7	29.3	14.7
	DAP- 23	2.7	6.4	4.7	23.4	23.6	27.5	11.6
	DAP- 29	4.1	11.2	10.7	27.9	22.5	18.0	5.6

DAP-R-L—Death Attitude Profile- Revised, DAP—number of item.

**Table 3:** The mean scores of the surveyed group of respondents for each scale of the DAP-R-PL questionnaire.

Death Attitude Profile-Revised	Average Answer Score of the Surveyed Respondents Group (Max. 7.0)
Fear of Death	5.3
Neutral Acceptance	5.3
Approach Acceptance	4.6
Escape Acceptance	4.4
Death Avoidance	3.9

Source: own study.

**Table 4:** Sub-scores of the Anxiety and Fascination with Death Scale (*N* = 516).

Anxiety and Fascination with Death Scale [%]		1 *	2	3	4
Fear of death subscale	Thinking about death depresses me (1)	5.0	38.4	43.6	13.0
	The prospect of my death is terrifying to me (3)	8.9	37.0	38.8	15.3
	I like to imagine how I will die (5)	54.8	35.5	7.6	2.1
	In moments of severe depression, I start thinking about my death (7)	44.4	40.9	13.2	1.6
	In a really tough situation I might decide to commit suicide (9)	52.9	33.9	11.4	1.7
	I think about death without fear (15)	21.1	45.3	30.0	3.5
	I don’t like movies in which one of the main characters dies (17)	10.9	39.9	35.4	13.8
	Sometimes I find it hard to tear myself away from the thought of death (18)	36.2	48.8	13.4	1.6
	Sometimes I imagine what will happen to me after death (20)	28.9	40.5	26.2	4.5
Death fascination subscale	I often wonder in what situation I would be willing to take my own life (2)	49.8	35.3	13.0	1.9
	Death is something unpleasant for me (4)	2.3	14.0	55.0	28.7
	When someone brings up the issue of death in my presence I quickly try to change the topic of conversation (6)	21.3	58.1	15.9	4.7
	I think I will pray more often as the years go by (8)	12.0	33.9	43.8	10.3
	I often think about death (10)	39.7	49.4	9.7	1.2
	I like to imagine my own funeral (11)	63.8	29.8	4.8	1.6
	I often think that I would like to die (12)	64.9	30.0	4.3	0.8
	It terrifies me, when people close to me dies (13)	3.1	13.4	40.2	43.2
	Death is a fascinating mystery to me (14)	23.8	41.1	29.7	5.4
	Death is the worst misfortune in human life (16)	14.7	43.2	29.1	13.0
	I enjoy learning about various rituals and ceremonies accompanying death (19)	34.5	43.2	19.4	2.7
	I like to meditate on topics related to death (21)	35.1	51.0	11.8	2.1
	I am interested in paintings depicting death and dying (22)	51.9	38.0	8.9	1.2
	I often talk to people about death (23)	25.5	56.0	15.9	2.9

* 1—definitely not, 2—rather not, 3—rather yes, 4—definitely yes. Source: own study.

**Table 5:** Influence of respondents’ sociodemographic factors on attitudes toward death.

Variables		Factors				
		Age	Gender	Marital Status	Place of Residence	Education
Death Attitude Profile- Revised	Fear of Death	0.0369	0.2487 *	0.0064	0.0896	0.0032
	Death Avoidance	−0.2147 *	−0.1145	−0.0478	−0.1279	−0.0058
	Neutral Acceptance	0.3412 *	−0.2637 *	0.2875 *	0.1097	−0.0654
	Approach Acceptance	0.0874	−0.1674	−0.1112	−0.2206 *	0.1010
	Escape Acceptance	−0.1245	0.1022	0.1236 *	0.4213	0.1365
Death Anxiety and Fascination Scale	Death anxiety	0.0125	−0.1845	0.1456	−0.1566	0.1456
	Death fascination	0.1478	0.1666	0.1269	−0.0987	−0.1789

*p* < 0.05. Pearson correlation * *p* < 0.05.

**Table 6:** Correlation coefficients of the seniority effect on nurses’ attitudes toward death.

Test Variable		Correlation Coefficient (r, *p* < 0.005)
Death Attitude Profile-Revised	Fear of Death	0.0865
	Death Avoidance	0.1355
	Neutral Acceptance	0.0361
	Approach Acceptance	0.0420
	Escape Acceptance	0.2143 ^#^
Death Anxiety and Fascination Scale	Death anxiety	0.0569
	Death fascination	0.0785

^#^*p* < 0.005.

## Data Availability

Not applicable.

## Supplementary Materials

The following supporting information can be downloaded at: www.mdpi.com/article/10.3390/ijerph192013119/s1. Table S1. Statements of the DAP-R-L.
