# Supplementary material for: Analysis of Nurses’ Attitudes toward Patient Death
Source: Int J Environ Res Public Health. 2022 Oct 12;19(20):13119. doi: 10.3390/ijerph192013119 (PMC9602489; doi:10.3390/ijerph192013119)
Supplement: Supplementary file 1 [file ijerph-19-13119-s001.zip › ijerph-1952929-supplementary.pdf]

Supplementary Materials

Table S1. Statements of the DAP-R-L- Death Attitude Profile- Revised

Item	Statements
DAP- 1	Death is no doubt a grim experience
DAP- 2	The prospect of my own death arouses anxiety in me
DAP- 3	I avoid death thoughts at all costs
DAP- 4	I believe that I will be in heaven after I die
DAP- 5	Death will bring an end to all my troubles
DAP- 6	Death should be viewed as a natural undeniable, and unavoidable event.
DAP-7	I am disturbed by the finality of death
DAP- 8	Death is an entrance to a place of ultimate satisfaction
DAP- 9	Death provides an escape from this terrible world.
DAP- 10	Whenever the thought of death enters my mind, I try to push it away.
DAP- 11	Death is deliverance from pain and suffering
DAP- 12	I always try not to think about death.
DAP- 13	I believe that heaven will be a much better place than this world.
DAP- 14	Death is a natural aspect of life.
DAP- 15	Death is a union with God and eternal bliss.
DAP- 16	Death brings a promise of a new and glorious life.
DAP- 17	I would neither fear death nor welcome it.
DAP- 18	I have an intense fear of death.
DAP- 19	I avoid thinking about death altogether.
DAP- 20	The subject of life after death troubles me greatly.
DAP- 21	The fact that death will mean the end of everything as I know it frightens me.
DAP- 22	I look forward to a reunion with my loved ones after I die.
DAP- 23	I view death as a relief from earthly suffering.
DAP- 24	Death is simply a part of the process of life.
DAP- 25	I see death as a passage to an eternal and blessed place.
DAP- 26	I try to have nothing to do with the subject of death.
DAP- 27	One thing that gives me comfort in facing death is my belief in the afterlife.
DAP- 28	I see death as a relief from the burden of this life.
DAP- 29	Death offers a wonderful release of the soul.
DAP- 30	Death is neither good nor bad.
DAP- 31	I look forward to a life after death.
DAP-32	The uncertainty of not knowing what happens after death worries me.
	DAP-R-L- Death Attitude Profile- Revised, DAP- number of item
